# 

**Published:** 1867-03

**Authors:** 


### CONTENTS.

### ORIGINAL CONTRIBUTIONS.

ART. XIV. LECTURE DELIVERED BY DR. J. BROWN, BEFORE THE MACON COUNTY MEDICAL SOCIETY, JANUARY 1, 1867, 129

ART. XV. POISONING BY STRYCHNIA—RECOVERY. By JULIEN S. SHERMAN, M.D., Chicago, 138

ART. XVI. THE SOCIAL EVIL. EXTENT OF PROSTITUTION AND ITS CONSEQUENCES IN NEW YORK CITY. PROPOSED LEGAL MEASURES TO MITIGATE ITS EVILS, 140

### CORRESPONDENCE.

ONIONS IN INTERMITTENT FEVER, 152

### SELECTIONS.

On Retrogressive Motions in Birds Produced by the Application of Cold to the Cervical Spine, with Remarks on the use of that Agent as an aid in Physiological Investigations. By S. Weir Mitchell, M.D., 154

The Permanganate of Potash in the Treatment of Carbuncle. By Thad. L. Leavitt, M.D., Germantown, Pa., 172

Remarks on Cancer in Connection with the Vital Nervous System and the Blood. Observations of Dr. Paul Broca, Surgeon l'Hospital Saint Antoine, Paris. By John O'Reilly, 175

### BOOK NOTICES.

The Renewal of Life; Lectures, chiefly Clinical. By Thomas K. Chambers, M.D., 179

The Functions and Disorders of the Reproductive Organs, in Childhood, Youth, Adult Age, and Advanced Life. By William Acton, M.R.C.S., 180

Guide for Using Medical Batteries. By Alfred C. Garratt, M.D., 181

A Vest-Pocket Medical Lexicon. By D. B. St. John Roosa, M.D., 181

Orthopædics: A Systematic Treatise upon the Prevention and Correction of Deformities. By David Prince, M.D., 182

### EDITORIAL.

Injustice to the Insane, 184

Delegates to the International Medical Congress in Paris, 184

Chicago Medical Society, 185

The Summer Course of Instruction, 185

Proceedings of Morgan County Medical Society, 186

The Body of Jeremy Bentham, 188

A Novel Method for the Production of Anatomical Plates, 188

Reopening of the Knoxville, Tenn., Deaf and Dumb Asylum, 153

Money Receipts, 189

